# Integrated Multiomics Analysis Identifies CDO1 as a Novel Therapeutic Target for Osteoarthritis

**DOI:** 10.2174/0118715303387442250918111006

**Published:** 2025-09-25

**Authors:** Zhihu Zhao, Xiangdong Wu, Duan Wang, Wei Luo, Jian-xiong Ma, Xin-long Ma

**Affiliations:** 1 Clinical School of Orthopedics, Tianjin Medical University, Tianjin, 300000, China;; 2 Department of Orthopaedics, Tianjin Hospital, Tianjin, 300000, China;; 3 Department of Orthopaedic Surgery, Beijing Jishuitan Hospital, Capital Medical University, Fourth Clinical College of Peking University, National Center for Orthopaedics, Beijing, 100035, China;; 4 Department of Orthopaedics, Orthopaedic Research Institute, West China Hospital, Sichuan University, Chengdu, 610041, China;; 5 Tianjin Institute of Orthopedics in Traditional Chinese and Western Medicine, No. 122, Munan Road, Tianjin, 300050, China

**Keywords:** Osteoarthritis, CDO1, single-cell transcriptome, mendelian randomization, cartilage structure, immunohistochemistry

## Abstract

**Introduction:**

This study aimed to identify key genes and potential therapeutic targets involved in the progression of osteoarthritis (OA) through an integrated multi-omics approach.

**Methods:**

We performed single-cell RNA sequencing (scRNA-seq) analysis on OA and control samples to define cell types and differentially expressed genes (DEGs). Bulk RNA-seq data from 7 public OA datasets were analyzed to identify DEGs, and Weighted Gene Co-expression Network Analysis (WGCNA) identified key co-expressed modules. An Integrated analysis of scRNA-seq DEGs, bulk RNA-seq DEGs, and WGCNA module genes pinpointed overlapping candidates. Functional enrichment analysis of these genes was then conducted. Mendelian randomization (MR) analysis was used to assess causal relationships between candidate genes and OA risk. The top candidate gene, CDO1, was functionally validated using siRNA-mediated knockdown in a rat OA model, assessed by histology and immunohistochemistry.

**Results:**

scRNA-seq identified 11 distinct cell types and 4,316 DEGs. Bulk RNA-seq meta-analysis revealed 3,664 DEGs, with WGCNA highlighting a key module significantly associated with OA. Integration identified 932 overlapping DEGs. Enrichment analysis implicated pathways including ferroptosis, PI3K-Akt signaling, and ECM-receptor interaction. MR analysis established CDO1 as the top causal OA risk gene (OR (95% CI) = 0.998 (0.996–0.999), *P* = 0.003).

**Discussion:**

*In vivo*, CDO1 knockdown significantly delayed OA progression in rats. Compared to controls, the si-CDO1 group showed improved cartilage structure, increased chondrocyte numbers, and enhanced type II collagen expression.

**Conclusion:**

CDO1 is a novel OA risk gene and therapeutic target. Its inhibition protects against OA progression, as supported by integrated multi-omics analysis and *in vivo* validation.

## 1 INTRODUCTION

Osteoarthritis (OA) has emerged as a significant public health concern, with its global burden increasing from 1990-2019, primarily due to population growth and aging [1-4]. OA is characterized by joint cartilage inflammation, making it the most common form of joint cartilage disease [5, 6]. It is more prevalent in women than in men, and its incidence has been further exacerbated by factors such as obesity, smoking, reduced physical activity, and joint injuries, which are more prevalent in modern lifestyles [7-10]. Globally, OA is a highly prevalent disabling disease that affects both physiological and psychological health and can even be a risk factor for some cardiovascular diseases [11, 12]. The treatment and rehabilitation of OA consume a substantial amount of medical resources [13]. As our society ages, OA has become a serious threat to public health and a major contributor to social and medical burdens [14, 15].

Over the past decade, traditional multiomics studies have focused primarily on understanding the molecular processes involved in OA progression [16, 17]. However, traditional multiomics sequencing technologies are limited because they can measure only the average expression levels of tens of thousands of cells together, thereby overlooking the cellular heterogeneity of chondrocytes in articular cartilage tissue [18-20].

With the advent of single-cell sequencing technology, it is now possible to study the specific cellular composition of degenerated or diseased tissues at an unprecedented resolution [21-25]. This technology has been increasingly applied to osteoarthritis-related tissues, such as cartilage and synovium [26-28]. Although a substantial amount of information has been obtained and extensively interpreted from these studies, many fundamental and physiologically significant insights remain unexplained due to the limitations of current analytical tools [29]. The advancement of data science, particularly in the field of bioinformatics, offers numerous analytical tools for interpreting data generated by single-cell sequencing [30]. These tools can help us uncover more detailed mechanisms of disease development, offering new perspectives and potential targets for therapeutic intervention.

Previous studies have applied this technique to OA, revealing differentially expressed genes (DEGs) in various cell types and identifying OA-specific cell clusters [31]. However, the exact roles of these DEGs in the initiation and progression of OA have not been well established. The causal effects of cellular DEGs on OA remain unconfirmed. To address this issue, Mendelian randomization (MR) has emerged as a valuable tool for estimating the causal effect of exposure on disease onset and prognosis [32, 33].

MR utilizes single-nucleotide polymorphisms (SNPs), which are genetic variants, as instrumental variables (IVs) for causal inference [34, 35]. These genetic variants are associated with risk factors but are independent of confounding factors, influencing the outcome only through these risk factors [36]. Based on these characteristics, the differences in outcomes between individuals carrying the variants and those who do not carry them can be attributed to differences in the risk factors [37, 38]. The associations derived using MR are less likely to be biased by reverse causation or potential confounding factors, providing more reliable insights into the causal relationships between exposures and outcomes [39].

However, in the context of DEG analysis, identifying genetic variants solely associated with a single component can be challenging. Gene expression quantitative trait loci (eQTLs) directly link gene expression to genetic variants, reveal regulatory mechanisms, reduce confounding effects, account for pleiotropy, improve the accuracy of causal inference, and support drug development. We further validated the results using *in vivo* samples. Thus, our study integrated scRNA-seq and MR analyses to test the hypothesis that differentially expressed genes may be causally associated with the risk of OA. By doing so, we aimed to clarify the potential causal relationships between cellular DEGs and the development of OA.

## 2 METHODS

### 2.1 Data Source and Preprocessing

In this study, we used the GSE255460 dataset, which includes single-cell RNA sequencing (scRNA-seq) data of human knee cartilage tissue from patients with OA (n=8) and non-OA controls (n=3). The scRNA-seq data were preprocessed using the Seurat package, which involves data normalization, identification of highly variable genes, data integration, data scaling, and principal component analysis (PCA). These steps ensured data quality and allowed the identification of genes with significant variability. Specifically, the data were normalized, and highly variable genes were selected for further analysis. Data from different batches were integrated using the FindIntegrationAnchors function with the selected highly variable genes as anchors. After integration, the data were scaled, and PCA was conducted to reduce dimensionality. UMAP was then applied for dimensionality reduction, followed by clustering analysis to identify distinct cell populations. To better understand the biological characteristics of each cluster, cell type annotations were performed based on known cell type information from prior studies [28]. After annotation, differential expression analysis was performed using the FindAllMarkers function to identify genes that were significantly different between patients with OA and healthy controls, with a log2-fold change (FC) greater than 0.5 and a false discovery rate (FDR) of less than 0.05. The differentially expressed genes were integrated into a unified list and visualized using a volcano plot. These genes, which were significantly differentially expressed between patients with OA and controls, may be closely associated with the pathological mechanisms of OA. The results were saved for further functional annotation and pathway analysis.

### 2.2 RNA Sequencing Analysis

The GSE51588, GSE55235, GSE55457, GSE57218, GSE82107, GSE98918, and GSE11799 datasets were downloaded from the Gene Expression Omnibus (GEO) database (https://www.ncbi.nlm.nih.gov/geo/). Gene expression data were log2-transformed for normalization to stabilize variance. Differential expression analysis was performed using the limma package (version 4.2.2) in R. Genes with an adjusted *P*-value < 0.05 and a log2-fold change (FC) > 2 were identified as differentially expressed genes (DEGs). Batch effects were removed using the 'removeBatchEffect' function from the limma package to reduce technical variations. The top 50 DEGs were visualized using a heatmap, which was generated with a dedicated heatmap package in R. To further explore the biological functions and pathways associated with these potential genes, Gene Ontology (GO) enrichment analysis and Kyoto Encyclopedia of Genes and Genomes (KEGG) pathway analysis were performed using the ClusterProfiler package (version 3.18.0) in R. Using the WGCNA method, an appropriate soft threshold β was selected to construct a scale-free network. Hierarchical clustering was performed using the dynamic tree cut method. Gene significance (GS) and module significance (MS) were calculated, and the module with the highest correlation coefficient was identified as the key module. The genes within this module were selected as candidate genes. We used R (version 4.2.2) for data analysis and constructed predictive models using the rms and rmda packages. These were primarily used to generate nomograms and calibration curves for evaluating the predictive performance of the models. Candidate genes were identified by intersecting the differentially expressed genes from single-cell analysis, the genes identified by the WGCNA model, and the differentially expressed genes obtained through RNA sequencing. The overlap among the three sources was visualized using a Venn diagram generated with the Venny online tool (https://bioinfogp.cnb.csic.es/tools/venny/).

### 2.3 MR Analysis

Mendelian randomization (MR) analysis was conducted using the TwoSampleMR R package (version 3.6.2, https://mrcieu.github.io/TwoSampleMR/) to investigate the causal relationships between genetic variants and OA [34]. Initially, single-nucleotide polymorphisms (SNPs) from previously published genome-wide association study (GWAS) data were extracted to serve as instrumental variables (IVs) for each trait [40, 41]. These SNPs were then clumped to obtain independent genetic variants, ensuring linkage disequilibrium (LD) r^2^ < 0.001 and a clumping distance of 1000 kb [42]. To select strong IVs, only SNPs with an F statistic greater than ten were considered, as this threshold is considered sufficient for predicting the exposures of interest.

The MR analysis process began by extracting the outcome data using the extract_outcome_data function from the TwoSampleMR package, ensuring that all relevant data for the outcome of interest were retrieved systematically [43]. The exposure and outcome data were subsequently harmonized using the harmonise_data function to ensure that the genetic variant exposure data matched the outcome data, correcting for potential inconsistencies between datasets (*e.g.*, strand mismatches).

For this analysis, only the inverse-variance weighted (IVW) method was employed to provide causal estimates [44]. The IVW method was selected because it offers robust and efficient estimates when the genetic variants used are valid instruments and there is no evidence of pleiotropy. Given that IVW assumes no pleiotropy and that the instrumental variables in this study met the criteria for valid instruments (with a sufficient F statistic), it was considered the most appropriate method for estimating causal effects between genetic variants and OA.

To ensure the accuracy and reliability of the results, estimates from the IVW method were calculated and visualized using forest plots [45]. These visualizations helped illustrate the causal effects of individual genetic variants on OA, allowing for the identification of key SNPs and providing insights into their potential causal role in OA development.

### 2.4 Establishment of a Rat OA Model

This study was approved by the Ethics Committee of Tianjin Hospital, Tianjin, China (YLS-2025-124). Sprague-Dawley rats (male; n = 40; 300±20 g) aged 3 months were purchased from Experimental Animal Centre of Tianjin Hospital and randomly assigned to four groups (n=10 per group) using a computer-generated randomization sequence: (1) Sham-operated group (joint capsule exposure only), (2) OA model group (anterior cruciate ligament (ACL) transection + meniscectomy + PBS injection), (3) si-CDO1 treatment group, and (4) control group (scrambled siRNA-loaded nanoparticles). All rats were anesthetized by intraperitoneal injection of 1% pentobarbital sodium (40 mg/kg). Rats were housed in IVC cages (22±1°C, 55% humidity, 12-hour light/dark cycle) with ad libitum access to food and water, as well as environmental enrichment (nesting material and PVC tubes). Once satisfactory anesthesia was achieved, the rats were fixed on the surgical table. The knee joint was shaved and prepared, followed by sterilization with an antiseptic solution. A 3 cm longitudinal incision was made along the medial side of the knee joint to expose the joint. A small incision was made along the medial edge of the patellar ligament, and the knee was flexed to evert the patella, exposing the joint cavity. The medial collateral ligament was cut using fine straight scissors, followed by removal of the medial meniscus and transection of the anterior cruciate ligament (ACL). A positive drawer test confirmed the transection of the ACL. After disinfection with iodine, the incision was sutured in layers. Postoperatively, penicillin was administered intramuscularly for 3 consecutive days to prevent wound infection. On the seventh day, 100 μL of PBS, si-CDO1 solution, or nanoparticle solution was injected into the joint cavity of each group for treatment. After 28 days of treatment, the animals were euthanized, and samples were collected to evaluate the effects of si-CDO1 in treating OA. Group allocation was concealed from surgeons during procedures. Histological assessments were performed by pathologists who were blinded to group assignments, using coded slides. Postoperatively, buprenorphine analgesia (0.05mg/kg SC q12h×48h) and penicillin prophylaxis (50,000 IU/kg IM×3d) were administered. Rats showing >20% weight loss or persistent lameness would be euthanized (no cases observed).

### 2.5 The Therapeutic Effect of si-CDO1 on OA

After the final gavage intervention, the rats were fasted for 12 hours. They were then anesthetized with an intraperitoneal injection of 1% pentobarbital sodium and euthanized by cervical dislocation. Cartilage tissue from the surface of the tibial plateau was collected, placed in 1.5 mL centrifuge tubes, and then stored at −80 °C for future use. The entire knee joint was dissected, and surrounding tissues were removed before being fixed in 40 g/L paraformaldehyde. After 48 hours of fixation, the joints were decalcified in EDTA and prepared for paraffin sectioning. Hematoxylin and eosin (H&E) staining was performed to evaluate cartilage morphology and cell distribution, safranin-O/fast green (S&F) staining was used to detect ACAN, and immunohistochemistry was used to assess COL-II production.

## 3 RESULTS

### 3.1 scRNA Results

The data before and after quality control are shown in Figs. (**1A** and **1B**), respectively. After quality control, a total of 80,352 cells and 19,063 genes were obtained and used for further analysis. The relationships between nCount_RNA and nFeature_RNA, nCount_RNA and percent.rb, and nCount_RNA and percent_mito after filtering are shown in Figs. (**1C**-**E**).

**Fig. (1):**
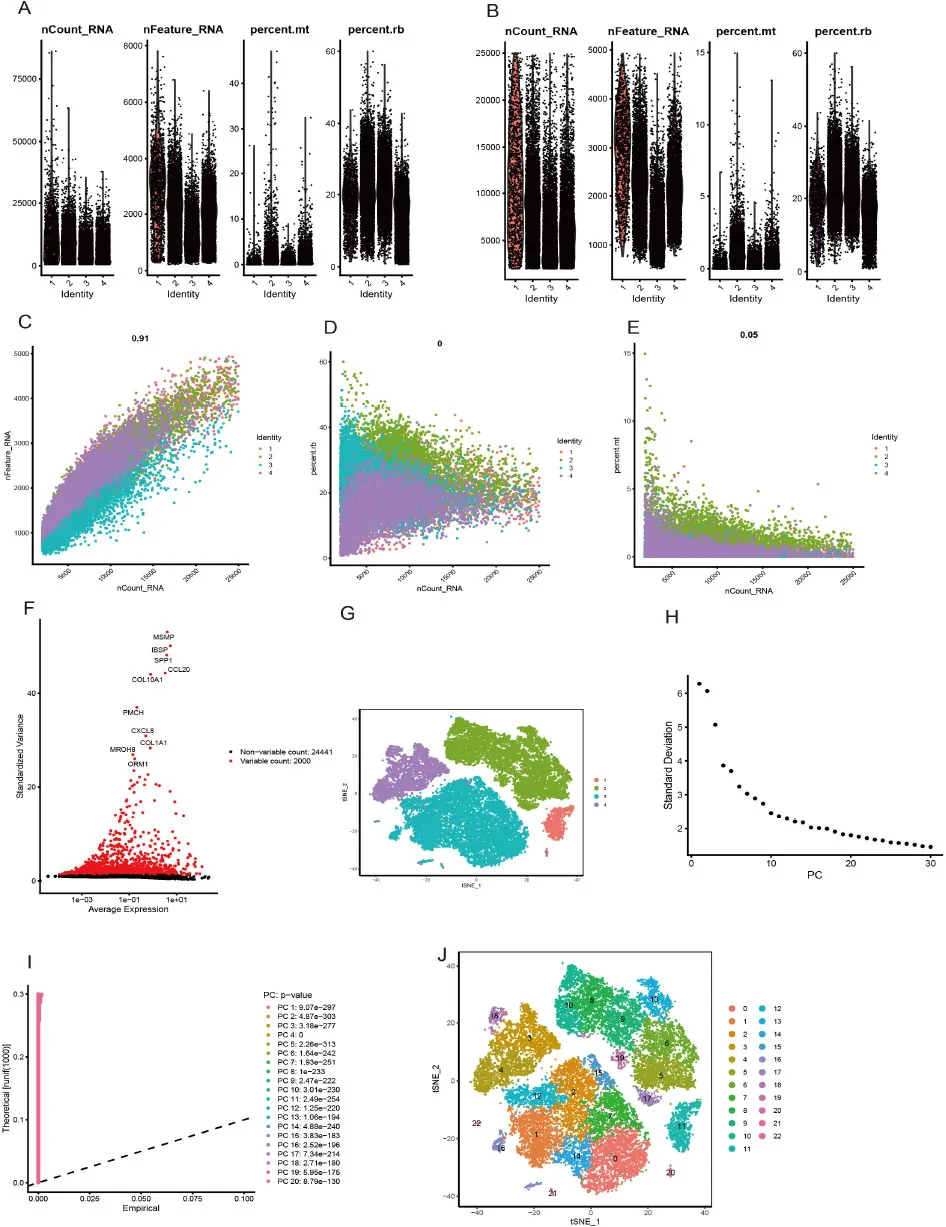
(**A**) Distribution of nFeature_RNA, nCount_RNA, and percent.mt before quality control. (**B**) Distribution of nFeature_RNA, nCount_RNA, and percent.mt after quality control. (**C**) Scatter plot showing the relationship between the number of genes detected (nFeature_RNA) and the number of RNA molecules (nCount_RNA) for each cell in single-cell RNA sequencing. (**D**) Scatter plot showing the relationship between the total number of RNA molecules (nCount_RNA) and the percentage of ribosomal RNA (percent.rb) for single-cell RNA sequencing. (**E**) Scatter plot showing the relationship between the total number of RNA molecules (nCount_RNA) and the percentage of mitochondrial RNA (percent.mt) in single-cell RNA sequencing. (**F**) Identification of 2000 hypervariable genes. (**G**) In total, 12 cell groups were obtained *via* TSNE clustering analysis (the resolution was 0.2). (**H**) The JackStraw method was used to screen the alternative dimensions. (**I**) The JackStraw method was used to screen the alternative dimensions. (**J**) Clusters were identified.

**Fig. (2):**
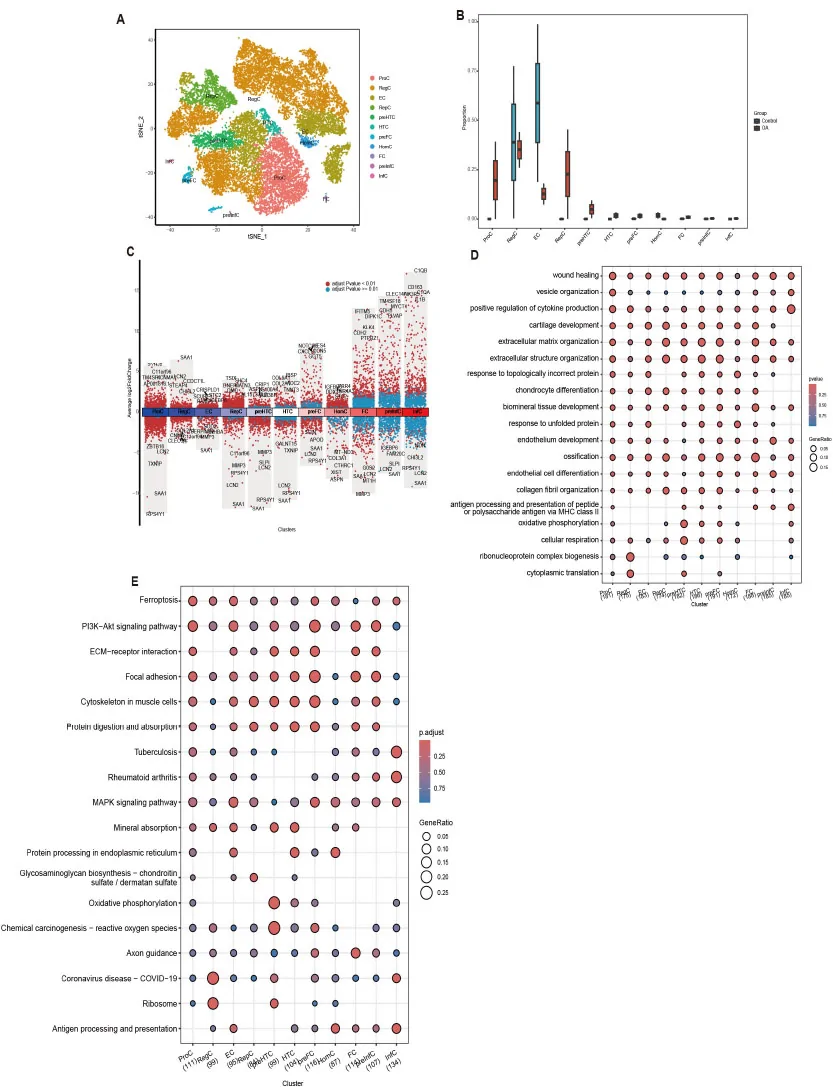
(**A**) Eleven cell subpopulations, including ProC, RegC, EC, RepC, preHTC, HTC, preFC, HomC, FC, preInfC, and InfC, were manually annotated. (**B**) Differences in the distributions of single-cell RNA sequencing subpopulations (ProC, RegC, EC, RepC, preHTC, HTC, preFC, HomC, FC, preInfC, and InfC) between patients with osteoarthritis (OA) and normal controls. (**C**) The top 5 upregulated and downregulated DEGs in each cluster. (**D**) Gene ontology of the differentially expressed genes in the OA and normal control groups. (**E**) KEGG pathway analysis of the differentially expressed genes between the OA and normal control groups.

The top 2000 highly variable genes were selected for cell type identification, and the top 10 were labeled (Fig. **1F**). The cell clusters were visualized using UMAP for dimensionality reduction (Fig. **1G**). The top 20 PCs were selected for analysis (*p* < 0.05) (Figs. **1H** and **1I**). A total of 23 clusters were included in the analysis (Fig. **1J**).To confirm the cellular heterogeneity of OA, we analyzed the scRNA-seq data from GSE255460, which included two OA tissues and two control tissues. Detailed information regarding the samples was reported previously. We first identified the cell clusters in UMAP (Fig. **2A**). We then compared the cell ratios between the two groups and identified 11 cell clusters: ProC, RegC, EC, RepC, preHTC, HTC, preFC, HomC, FC, preInfC, and InfC, as well as oligodendrocytes (Fig. **2B**). We next identified 4,316 DEGs (log2FC > 0.5) between the control and OA groups across 11 cell types, and all DEGs are shown in Fig. (**2C**). The biological processes included cytoplasmic translation, ribonucleoprotein complex biogenesis, cellular respiration and oxidative phosphorylation, antigen processing and presentation *via* MHC class II, collagen fibril organization and ossification, endothelial cell differentiation and endothelium development, response to unfolded and topologically incorrect proteins, extracellular matrix organization and cartilage development, and positive regulation of cytokine production and wound healing. The cellular components included the collagen-containing extracellular matrix, primary lysosome, membrane raft, membrane microdomain, endoplasmic reticulum lumen, apical part of the cell, ficolin-1-rich granule lumen, endocytic vesicle, ribosomal subunit, cytosolic ribosome, inner mitochondrial membrane protein complex, fibrillar collagen trimer, axonal growth cone, and MHC class ll protein complex. The molecular functions included protein sequestering activity, kinase activator activity, cadherin binding, extracellular matrix structural constituent conferring tensile strength, integrin binding, extracellular matrix structural constituent, glycosaminoglycan binding, MHC class II protein complex binding, MHC protein complex binding, ATP−dependent protein folding chaperone, growth factor binding, protein folding chaperone, structural constituent of ribosome, rRNA binding and platelet−derived growth factor binding (Fig. **2D**). The enriched KEGG pathways included ferroptosis, the PI3K–Akt signaling pathway, ECM–receptor interaction, focal adhesion, regulation of the cytoskeleton in muscle cells, protein digestion and absorption, tuberculosis, rheumatoid arthritis, the MAPK signaling pathway, mineral absorption, protein processing in the endoplasmic reticulum, glycosaminoglycan biosynthesis (chondroitin sulfate/dermatan sulfate), oxidative phosphorylation, chemical carcinogenesis (reactive oxygen species), axon guidance, coronavirus disease (COVID-19), ribosome biogenesis, and antigen processing and presentation (Fig. **2E**).

### 3.2 Bulk RNA Results

**Fig. (3):**
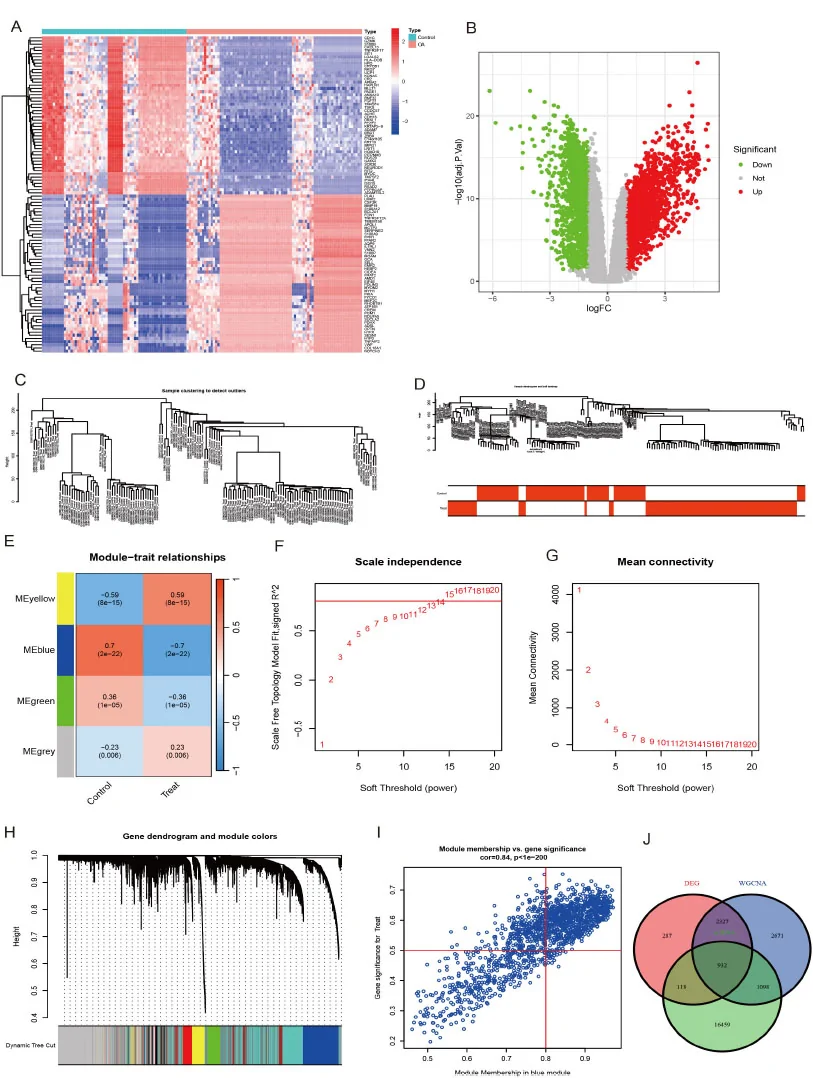
WGCNA was performed to identify gene modules highly correlated with OA and to identify candidate hub genes for functional analysis. (**A**) Volcano plot of genes that were differentially expressed between the OA and control groups. (**B**) Heatmap of differentially expressed genes (top 10 upregulated genes and downregulated genes) between the OA and control groups. (**C**) Sample hierarchical clustering. (**D**) Sample hierarchical clustering and heatmap of clinical traits. (**E**) Soft threshold screening. (**F**) Identification of coexpression modules. (**G**) Identification of mean connectivity. (**H**) Heatmap of the correlations of modules and phenotypes. (**I**) Scatter plot of correlations between modules and genes. (**J**) Identification of intersection marker genes.

Ultimately, GSE51588, GSE55235, GSE55457, GSE57218, GSE82107, GSE98918 and GSE117999 were included for analysis. The OA group consisted of 125 subjects, while the control group comprised 66 individuals. A total of 3664 DEGs were identified between the OA and control samples, including 1893 downregulated and 1771 upregulated genes in the OA samples (Figs. **3A** and **B**). WGCNA was performed, and no outlier samples were detected (Fig. **3C**). Sample clustering with clinical traits is shown in Fig. (**3D**). A heatmap was constructed to show the correlations between gene modules (MEYellow, MEBlue, MEGreen, and MEGrey) and traits. The yellow module indicates strong positive correlations with the trait, the blue module indicates moderate correlations, the green module indicates weak correlations, and the grey module indicates no significant correlations (Fig. **3E)**. A plot showing the scale-free topology fit index (R^2^) *versus* the soft threshold power was also constructed. The optimal power value was 8, where R^2^ was maximized, suggesting the best network construction parameter (Fig. **3F**). The mean connectivity of the network, varying with different soft threshold powers, was graphed to help select the optimal power that balances network connectivity (Fig. **3G**). Four modules were subsequently obtained by the dynamic tree-cutting algorithm (Fig. **3H**). The blue module, containing 7028 genes, was subsequently selected as the OA-related module based on the results of the module-trait correlation analysis (Fig. **3I**). A total of 932 DEGs were acquired by overlapping 3664 DEGs between OA and control samples, 7028 key genes from WGCNA, and 18,607 DEGs from scRNA-seq analysis (Fig. **3J**).

### 3.3 GO and KEGG Pathway Analyses

**Fig. (4):**
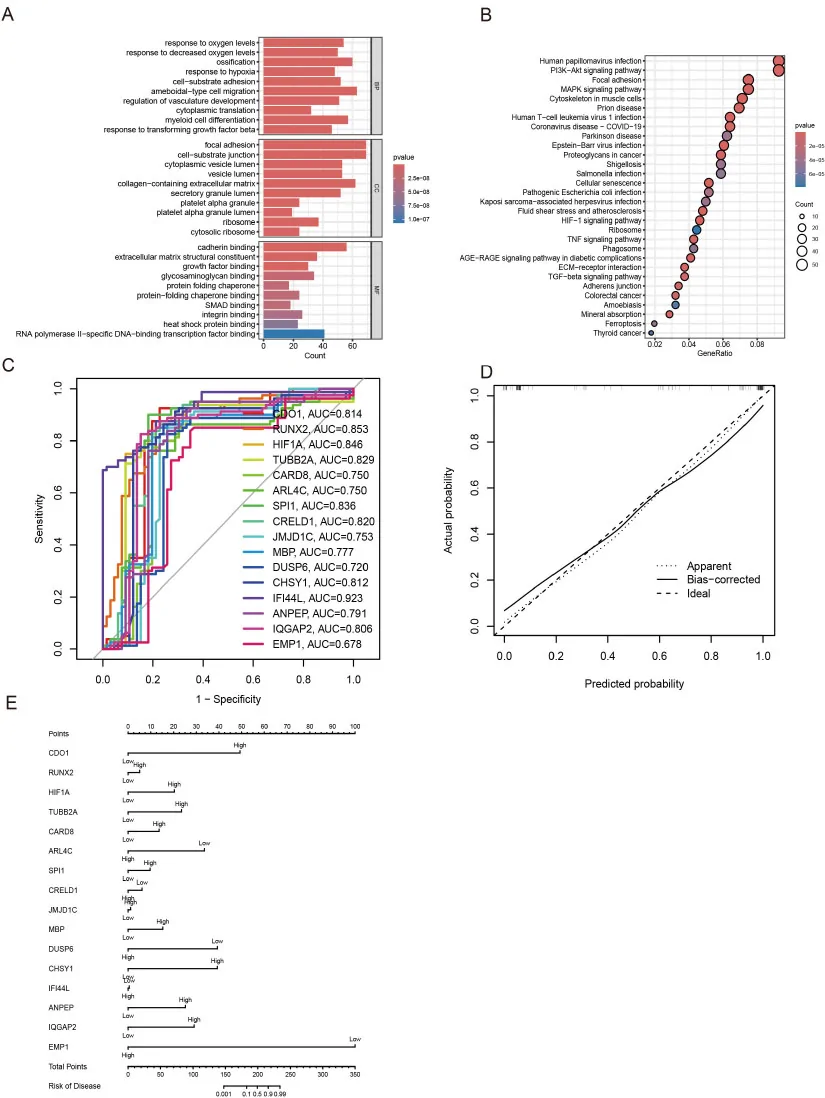
**A** Gene Ontology of the differentially expressed genes (DEGs). (**B**) KEGG pathway analysis of the differentially expressed genes. (**C**) ROC curves of feature genes (GSE56116). (**D**) Calibration curve for the nomogram model. (**E**) Nomogram for predicting osteoarthritis.

Biological processes were enriched mainly in responses to oxygen and decreased oxygen levels, ossification, response to hypoxia, cell–substrate adhesion, amoeboid-type cell migration, regulation of vasculature development, cytoplasmic translation, myeloid cell differentiation, and response to transforming growth factor-β (Fig. **4A**). The enriched cellular components included focal adhesion, cell–substrate junction, cytoplasmic vesicle lumen, vesicle lumen, collagen-containing extracellular matrix, secretory granule lumen, platelet α-granule, platelet α-granule lumen, ribosome, and cytosolic ribosome (Fig. **4A**). The main enriched molecular functions included cadherin binding, extracellular matrix structural constituent, growth factor binding, glycosaminoglycan binding, protein folding chaperone, protein folding chaperone binding, SMAD binding, integrin binding, heat shock protein binding, and RNA polymerase II-specific DNA-binding transcription factor binding (Fig. **4A**).

The enriched KEGG pathways included human papillomavirus infection, the PI3K–Akt signaling pathway, focal adhesion, the MAPK signaling pathway, cytoskeleton in muscle cells, prion disease, human T-cell leukemia virus 1 infection, coronavirus disease (COVID-19), Parkinson disease, Epstein‒Barr virus infection, proteoglycans in cancer, shigellosis, Salmonella infection, cellular senescence, pathogenic *Escherichia coli* infection, Kaposi sarcoma-associated herpesvirus infection, fluid shear stress and atherosclerosis, the HIF-1 signaling pathway, ribosome, the TNF signaling pathway, the AGE‒RAGE signaling pathway in diabetic complications, phagosome, ECM‒receptor interaction, the TGF‒beta signaling pathway, adherens junction, colorectal cancer, amoebiasis, mineral absorption, ferroptosis and thyroid cancer (Fig. **4B**).

Fig. (**4C**) presents the ROC curves for multiple genes used to assess their diagnostic performance for OA, with AUC values ranging from 0.678 (EMP1) to 0.923 (IFI44L), highlighting varying predictive capabilities across genes such as RUNX2 (0.853), SPI1 (0.836), HIF1A (0.846), CRELD1 (0.820), TUBB2A (0.829), CDO1 (0.814), IQGAP2 (0.806), CHSY1 (0.812), DOCK1 (0.814), MBP (0.777), ANPEP (0.791), CARD8 (0.750), ARL4C (0.750), JMJD1C (0.753), and DUSP6 (0.720).

Fig. (**4D**) shows the calibration curve for the predictive model, comparing the predicted and actual probabilities. The bias-corrected line is closely aligned with the ideal line, particularly in the mid-range of the predicted probabilities (0.2–0.8), indicating good model calibration. However, slight deviations were observed at the extremes (near 0 and 1), suggesting minor discrepancies in prediction accuracy for low and high probabilities. In addition, a nomogram prediction model was constructed by integrating the expression levels of these genes (CDO1, RUNX2, HIF1A, TUBB2A, CARD8, ARL4C, SPI1, CRELD1, JMJD1C, MBP, DUSP6, CHSY1, IF44L, ANPEP, IQGAP2, and EMP1) to predict disease risk (Fig. **4E**).

### 3.4 MR Results

**Fig. (5):**
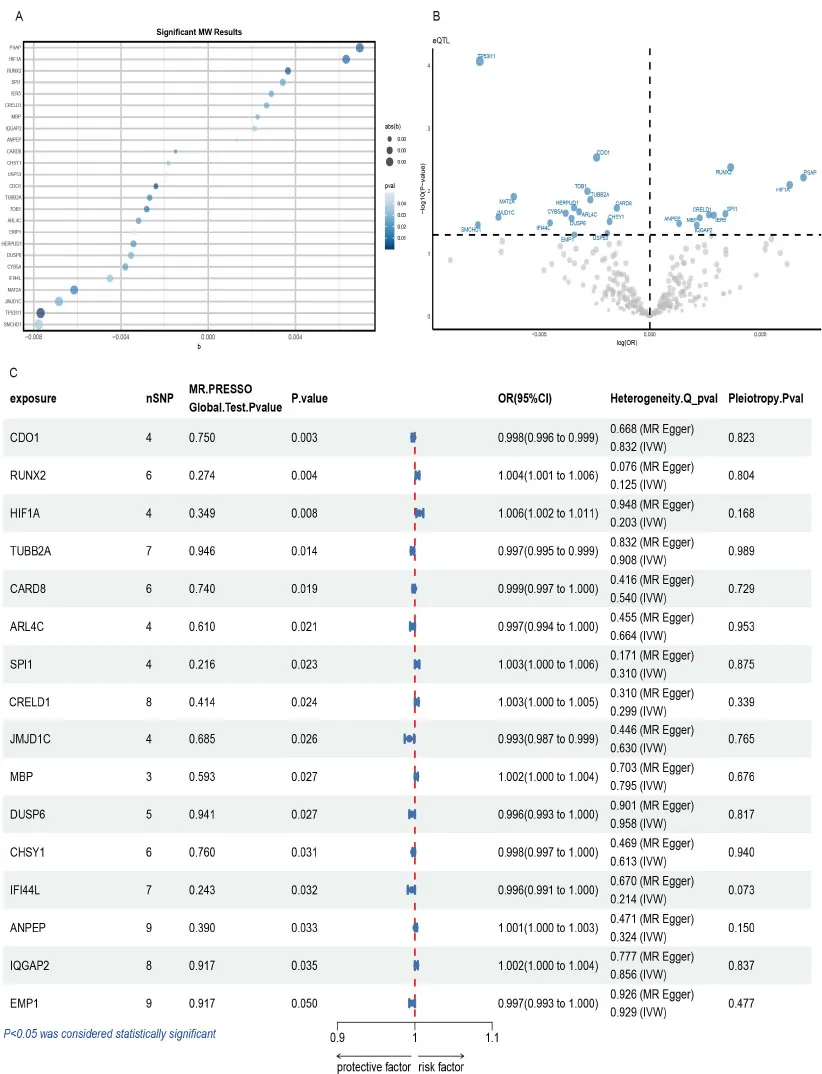
(**A**) Lollipop plot showing all MR results for each DEG. The X-axis represents the R coefficient of correlation. The Y-axis represents each DEG in osteoarthritis. The color scale indicates the *p*-value. (**B**) Bubble plot of the hub genes (eQTLs) associated with osteoarthritis. (**C**) Forest plot of the hub genes (eQTLs) associated with osteoarthritis.

To further investigate the roles of these DEGs, we extracted the eQTLs (genetic variations) of these 932 DEGs from the GSWA summary website as exposure variables and ischemic stroke (eqtl-a-ENSG00000100644) as the outcome variable for the two-sample MR analysis. We found that 9 DEGs were positively associated with the outcome, and 16 DEGs were negatively correlated with the outcome (Figs. **5A** and **B**). Among the 25 DEGs, CDO1 was identified as the top risk factor for osteoarthritis. However, its role in ischemic stroke has not been fully investigated. Given the best causal estimation, elevated levels of CDO1 were significantly associated with an increased risk of OA (OR (95% CI) = 0.998 (0.996–0.999), *P*= 0.003; Fig. **5C**).

### 3.5 Knockdown of CDO1 Delayed OA Progression

**Fig. (6):**
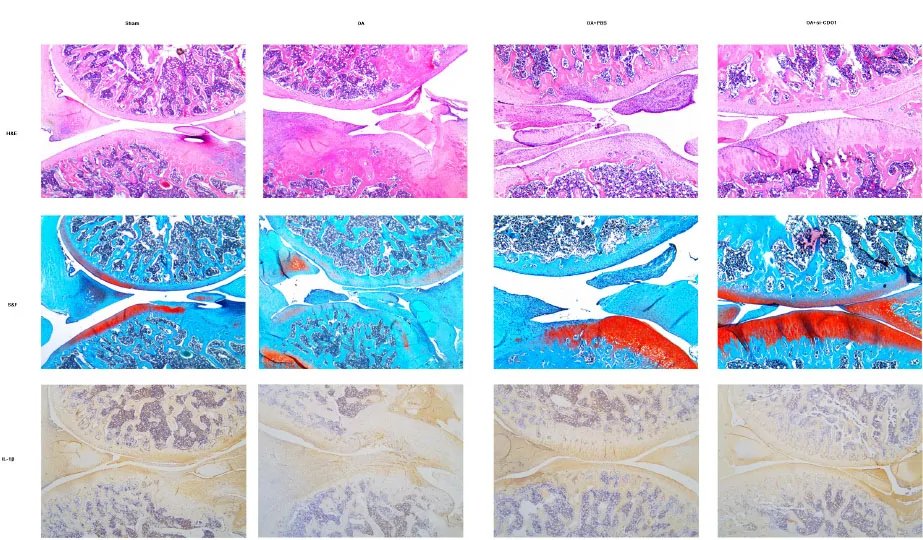
Hematoxylin and eosin (H&E) staining, safranin-O/fast green (S&F) staining, and immunohistochemical analysis of the production of COL-II.

To further verify the role of CDO1 in OA, si-CDO1 was injected after the medial collateral ligament and meniscus were transected. The effects of si-CDO1 on delaying OA progression were observed through HE staining, Safranin O/Fast Green staining, and type II collagen immunohistochemistry. The results revealed that the cartilage surface in the sham-operated group was smooth, with normal tissue morphology and a single-row distribution of chondrocytes. In comparison, the model group presented an irregular cartilage surface, cartilage tissue defects, thinning of the cartilage layer, a clustered distribution of chondrocytes, and a reduced number of chondrocytes. After si-CDO1 treatment, the cartilage surface became smooth, the cartilage layer significantly thickened, and the number of chondrocytes increased, with an orderly arrangement. In the sham-operated group, the chondrocytes exhibited blue-purple nuclei, and proteoglycan staining in the cartilage matrix was uniform, appearing blue or blue-purple, with an intact tidemark. In the model group, the number of chondrocytes was reduced, proteoglycan staining in the cartilage matrix was decreased, and the tidemark was incomplete. Compared with the model group, the si-CDO1 group exhibited an increased number of chondrocytes and deeper staining of the cartilage matrix. However, the staining was slightly lighter than that in the sham-operated group. The results of type II collagen immunohistochemical staining revealed varying degrees of type II collagen expression in the cartilage tissue of the knee joints in all groups, with brownish-yellow granules observed in the cytoplasm. Compared with the sham-operated group, the OA group presented a significant reduction in the number of ADAMTS5-positive cells and lighter staining. Compared with the model group, the si-CDO1 group presented an increase in the number of type II collagen-positive cells and slightly deeper staining (Fig. **6**). These results suggest that si-CDO1 can prevent the onset and progression of OA.

## 4 DISCUSSION

Currently, few studies have combined scRNA-seq, RNA-seq, and Mendelian randomization, and we applied this approach to estimate the causal associations between hub genes and the risk of OA. Combining MR with scRNA-seq can provide valuable insights into causal relationships between genetic variants, gene expression, and complex traits, which is useful in comparing expression levels across cell types, examining expression patterns within specific cell populations, or evaluating the impact of genetic variants on gene expression heterogeneity. Our study demonstrated that CDO1 had a positive causal effect on the risk of OA, which was significantly causally associated with vessel infarction. Further *in vivo* study revealed that si-CDO1 can prevent the onset and progression of OA.

Our findings from the scRNA-seq analysis were robust and consistent with previous reports, which revealed OA immune cell heterogeneity [28]. Fan *et al.* [28] identified InfC, preHTC, preFC, and HTC as potential cell populations for targeting in OA therapy. Among the 2,339 DEGs, we applied MR analysis and identified 58 genes potentially involved in OA development. These genes were distributed across multiple cell populations, including chondrocytes, synovial cells, and immune cells. Pathway enrichment analysis revealed that these genes are significantly involved in biological processes such as extracellular matrix organization, the inflammatory response, and angiogenesis, consistent with the multifactorial etiology of OA [46-48].

CDO1 is a key rate-limiting enzyme in the cysteine catabolic pathway [49-51]. Its core function is to catalyze the oxidation of cysteine into cysteine sulfinic acid, thereby significantly depleting the limited intracellular pool of free cysteine [52]. Given that cysteine is an indispensable and often rate-limiting precursor amino acid for glutathione synthesis, the active expression of CDO1 directly constrains GSH biosynthesis [51-53]. GSH is one of the most crucial intracellular antioxidants, particularly serving as an essential cofactor for glutathione peroxidase 4 (GPX4) [54]. The primary function of GPX4 is to scavenge harmful lipid peroxides [55]. Consequently, CDO1-mediated cysteine depletion leads to decreased GSH levels, which subsequently weaken GPX4 activity and cause a substantial accumulation of lipid peroxides [56]. This process, in the presence of iron ions, uncontrollably triggers an iron-dependent chain reaction of lipid peroxidation, ultimately inducing ferroptosis—a regulated form of cell death characterized by lipid peroxidation [57-61]. Within the complex pathological environment of OA, oxidative stress and ferroptosis play central roles in driving the disease [62]. Joint tissues, particularly chondrocytes and synovial cells, are chronically exposed to various stressors (such as mechanical injury and inflammatory factors), leading to the sustained generation of reactive oxygen species (ROS) [58, 59]. The potential activation of CDO1 further disrupts the already compromised antioxidant defense system (specifically the GSH-GPX4 axis), exacerbating oxidative damage [63]. In cartilage, this directly results in chondrocyte dysfunction, senescence, ferroptosis, and apoptosis, and induces catabolic enzymes that accelerate the degradation of the extracellular matrix. In the synovium, damage-associated molecular patterns (DAMPs) released due to oxidative stress and ferroptosis can potently activate innate immune responses, initiating and sustaining chronic synovitis and promoting the release of inflammatory factors, thereby further disrupting joint homeostasis [64-67].

The significant finding of this study suggests that under OA pathological conditions, the downregulation of CDO1 expression may exert a protective effect. The underlying mechanism lies in the fact that reduced CDO1 activity helps maintain the intracellular availability of cysteine, thereby ensuring sufficient GSH synthesis. Elevated GSH levels effectively support GPX4 activity, enhancing the cell’s capacity to clear lipid peroxides and other ROS, and significantly mitigating oxidative stress damage [68]. More critically, maintaining the integrity of the GSH-GPX4 axis is essential for inhibiting ferroptosis [69-71]. Therefore, CDO1 downregulation, by stabilizing intracellular redox balance and blocking the ferroptosis pathway, likely exerts protective effects at multiple levels: protecting chondrocyte survival and function, reducing matrix degradation, and suppressing synovial inflammation. Collectively, these effects contribute to delaying the pathological progression of OA, including cartilage destruction, osteophyte formation, and symptom exacerbation.

Notably, CDO1 showed particularly strong potential in our study, demonstrating consistent causal associations across multiple analyses. Its role in redox biology and vascular pathology suggests that it has dual contributions to OA progression, directly influencing cartilage homeostasis and modulating immune-mediated inflammatory responses. Additionally, the causal link identified between CDO1 and vascular occlusion further supports its broader impact on vascular health, which is increasingly recognized as a key factor in OA progression [72].

OA is a chronic degenerative disease involving complex interactions of mechanical, inflammatory, and genetic factors [73-77]. Despite this complexity, the methodological similarities highlighted in our study underscore the diversity and broad applicability of integrating scRNA-seq and MR for identifying disease targets.

This study has several limitations: the potential influence of horizontal pleiotropy in MR analysis, the need for further validation of the findings in independent cohorts, and the limited examination of environmental factors and their interactions with genetic susceptibility.

## CONCLUSION

Our combined analyses provide robust genetic evidence supporting a positive causal association between elevated CDO1 levels and an increased risk of OA. *In vivo* experiments demonstrated that si-CDO1 treatment effectively delayed the progression of OA by preserving cartilage integrity and enhancing chondrocyte function. These findings suggest that targeting CDO1 could be a promising therapeutic strategy for the prevention and treatment of OA.

## Data Availability

All data generated or analyzed during this study can be obtained from the corresponding author upon reasonable request.

## LIST OF ABBREVIATIONS

ScRNA-Seq: Single-Cell RNA Sequencing
MR: Mendelian Randomization
OA: Osteoarthritis
DEGs: Differentially Expressed Genes
UMAP: Uniform Manifold Approximation and Projection
WGCNA: Weighted Gene Co-Expression Network Analysis
SNPs: Single-Nucleotide Polymorphisms
IVs: Instrumental Variables
eQTLs: Expression Quantitative Trait Loci
PCA: Principal Component Analysis
FC: Fold Change
FDR: False Discovery Rate
GEO: Gene Expression Omnibus
GO: Gene Ontology
KEGG: Kyoto Encyclopedia of Genes and Genomes
GS: Gene Significance
MS: Module Significance
GWAS: Genome-Wide Association Study
LD: Linkage Disequilibrium
IVW: Inverse-Variance Weighted
ACL: Anterior Cruciate Ligament
H&E: Hematoxylin and Eosin
S&F: Safranin-O/Fast Green
GPX4: Glutathione Peroxidase 4
ROS: Reactive Oxygen Species
DAMPs: Damage-Associated Molecular Patterns
